# Oestrogen deficiency modulates particle-induced osteolysis

**DOI:** 10.1186/ar3381

**Published:** 2011-06-22

**Authors:** Christophe Nich, Jean Langlois, Arnaud Marchadier, Catherine Vidal, Martine Cohen-Solal, Hervé Petite, Moussa Hamadouche

**Affiliations:** 1 Laboratoire de Bioingénierie et Biomécanique Ostéo-articulaires, Faculté de Médecine Paris 7-Denis Diderot, 10, avenue de Verdun, 75010 Paris, France; 2 Useful Progress, 23, rue d'Anjou, 75008 Paris, France; 3 Institut Pasteur and INSERM U747, Laboratory of Stem cells, Signaling and Prions, Université Paris 5-René Descartes, 45 Rue des Saints-Pères, 75270 Paris Cedex 06, France; 4 INSERM U606, Faculté de Médecine Paris 7-Denis Diderot, 10, avenue de Verdun, 75010 Paris, France

## Abstract

**Introduction:**

Postmenopausal osteoporosis may modulate bone response to wear debris. In this article, we evaluate the influence of oestrogen deficiency on experimental particle-induced osteolysis.

**Methods:**

Polyethylene (PE) particles were implanted onto the calvaria of normal controls, sham-ovariectomized (OVX), OVX mice and OVX mice supplemented with oestrogen (OVX+E). After 14 days, seven skulls per group were analyzed using a high-resolution micro-computed tomography (micro-CT) and histomorphometry, and for tartrate-specific alkaline phosphatase. Five calvariae per group were cultured for the assay of IL-1β, IL-6, TNF-α and receptor activator of the nuclear factor κB (RANKL) secretion using quantitative ELISA. Serum IL-6 concentrations were obtained. The expression of RANKL and osteoprotegerin (OPG) mRNA were evaluated using real-time PCR.

**Results:**

As assessed by μCT and by histomorphometry, PE particles induced extensive bone resorption and an intense inflammatory reaction in normal controls, sham-OVX and OVX+E mice, but not in the OVX mice group. In normal controls, sham-OVX and OVX+E mice, PE particles induced an increase in serum IL-6, in TNF-α and RANKL local concentrations, and resulted in a significant increase in RANKL/OPG messenger RNA (mRNA) ratio. Conversely, these parameters remained unchanged in OVX mice after PE implantation.

**Conclusions:**

Oestrogen privation in the osteolysis murine model ultimately attenuated osteolytic response to PE particles, suggesting a protective effect. This paradoxical phenomenon was associated with a down-regulation of pro-resorptive cytokines. It is hypothesized that excessive inflammatory response was controlled, illustrated by the absence of increase of serum IL-6 in OVX mice after PE implantation.

## Introduction

Aseptic loosening of total joint replacements develops as a consequence of periprosthetic osteolysis, caused by a macrophage-mediated inflammatory reaction [1,2]. Although it is well established that generation of polyethylene (PE) particles by the bearing couple is correlated with the risk for revision due to aseptic loosening [3], great variations in the degree of osteolysis are sometimes observed in clinical practice. This suggests that patient-related factors cause variability in the host response to PE particles. Authors have reported various factors, such as genetically determined obesity [4], and genetic background [5], as potential candidates involved in the modulation of host response to PE particles. In a recent work, we evaluated the bone response to particulate debris in an ovariectomized mice model [6]. We hypothesized that high bone turnover driven by oestrogen deficiency following ovariectomy would result in accelerated bone loss in the area in contact with the PE particles. Paradoxically, particle-induced osteolysis was found significantly decreased in this model, indicating that ovariectomy could be protective against wear debris-induced osteolysis, possibly via sex steroid hormone deficiency. Particle-induced osteolysis has been shown to follow a biologic pathway, driven by cytokines such as interleukin (IL)-1β, IL-6, and tumor necrosis factor (TNF)-α. These mediators have been found in the soft tissue of joints with loosened prostheses [7] and appear to be released by human macrophage or macrophagelike cells *in vitro *[8]. There is evidence that the RANK (receptor activator of the nuclear factor κB)/RANKL (RANK ligand) system acts downstream from IL-1, IL-6 and TNF-α and may be the ultimate effector mediating the effects of cytokines on the regulation of bone resorption [9]. Ovariectomy has been shown to decrease bone mass, stimulate bone remodeling, and increase IL-6 levels in bone marrow [10,11], and to produce an osteoporotic bone phenotype, similar to postmenopausal osteoporosis. Interestingly, high levels of the three above-mentioned cytokins have been found in bone deprived of oestrogen [12].

Although the osteoprotective action of oestrogen is demonstrable in rodents, its implication in particle-induced osteolysis has never been shown. The purpose of the present study was to investigate the influence of oestrogen on the mechanism underlying particle-induced osteolysis in the murine calvaria model. Following our previous findings, we primarily hypothesized that oestrogen supplementation in ovariectomized mice would restore an extensive bone response to PE particles. In an attempt to shed light on possible underlying regulation, we secondly evaluated the local implication of the RANK/RANKL/OPG system and IL-1β, IL-6, TNF-α cytokins.

## Materials and methods

### Particles

Pure polyethylene particles (Ceridust VP 3620) were obtained from Clariant (Gersthofen, Germany). In our hands, the particle size was determined using scanning electron microscopy as previously detailed [6]. The mean particle size was 5.14 μm ± 3.05 μm (median 4.39 μm; range 0.52 to 15.5 μm). More than 55% of the particles were less than 5 μm in size. All particles were washed in ethanol to remove endotoxins, and then dried in a dessicator [6]. This treatment resulted in negative testing for endotoxins using a quantitative Limulus Amebocyte Lysate (LAL) Assay (Charles River, Margate, UK) at a detection level of <0.05 EU/mL. Particles were stored at 4°C before implantation.

### Animal surgery and experimental protocol

Eleven week-old C57BL/6J female mice (*n *= 96) were purchased from Janvier Laboratory (Le Genest-Saint-Isle, France). All mice were handled in agreement with French and international guidelines for care and use of laboratory animals. The protocol was given ethical approval by the local Animal Care Committee. Forty-eight mice were subjected to bilateral ovariectomy (OVX group) via the dorsal approach under general anesthesia (isoflurane). Twenty-four mice were sham-ovariectomized (sham-OVX), that is, ovaries were exteriorized, but not removed. Twenty-four non-OVX mice constituted the normal control group. Animals were housed in quarantine under local vivarium conditions (24°C and 12 h/12 h light/dark cycle) for one week.

Surgical implantation of PE particles has been previously described [4]. Briefly, mice were operated on under general anesthesia via inhaled isoflurane. All animals were 12 weeks old at surgery. A 0.5 × 0.5 cm^2 ^area of periostum was exposed by making a 10 mm midline sagittal incision over the calvaria. In PE-implanted groups, the particle powder (20 μg) was distributed uniformly over the intact periosteum using a sterile sharp surgical spoon. Table 1 shows the treatment of the different experimental groups of mice. Postoperatively, 24 OVX animals, including 12 sham-implanted mice and 12 mice implanted with particles, were given subcutaneous oestrogen replacement therapy five days per week (15 μg 17 beta-estradiol/kg body weight/day, Sigma Inc., St. Louis, MO, USA), and 24 OVX animals, including 12 sham-implanted mice and 12 mice implanted with particles, received subcutaneous vehicle only. Water and food were given *ad libitum*. Body weights were measured weekly, and the E injections were adjusted accordingly. Fourteen days postoperatively, the animals were sacrificed by an overdose of intraperitoneal sodium pentobarbital. The uteri were harvested and weighted to confirm oestrogen loss.

**Table 1:** Experimental mice groups

	**Normal control group**	**Sham-OVX group**	**OVX group**		**Total (n)**
				
			**OVX + Vehicle**	**OVX+E**	
	
PE (-)	12	12	12	12	48
PE (+)	12	12	12	12	48
Total (n)	24	24	24	24	96

E, oestrogen; PE(-), sham-implanted; PE(+), PE-implanted; OVX, ovariectomy.

### Micro-computed tomography imaging and volumetric osteolysis analysis

Seven calvariae per group were dissected after sacrifice. Specimens were freed of all soft tissues, and fixed in 4% neutralized paraformaldehyde. The skulls were analyzed with a high-resolution micro-computed tomograph (micro-CT) (Skyscan 1172; Skyscan, Aartselaar, Belgium) to perform qualitative and quantitative analyses of calvarial bone. Imaging analysis mainly focused on the osseous properties in the area of the skull sagittal suture. The radiographic projections (*n *= 210) were acquired at 80 kV and 100 μA with an exposure time of 100 ms. Eight frames were averaged for each rotation increment of 0.9° to increase the signal to noise ratio. 3D images were reconstructed with a voxel average size of 10 μm, using the manufacturer reconstruction software (NRecon, Skyscan). Qualitative and quantitative data were analyzed with a global fixed threshold [13]. To minimize the bias from the 3D orientation of the calvaria, a spheric volume of interest (VOI of 2 mm diameter) with the midline suture of the skull in its center was defined, as previously described [6]. For quantitative analysis of PE particle-induced osteolysis, the resident software (CTAn, Skyscan) was used to obtain the following measurements within the VOI: bone volume (BV, mm^3^), and trabecular thickness (Tb.Th., mm). To address bone tissue mineralization following oestrogen depletion/substitution, a relative density measurement was computed. For this purpose, the mean value of grey-level intensity, reflecting the density of bone tissue, was obtained in entire calvariae.

### Histologic evaluation of osteolysis

Calvariae were processed undecalcified after embedding in polymethyl-methacrylate according to a method previously described [6]. Sections (10 μm) were obtained at the depth where the presence of particles was detected within the calvaria tissue. These sections were stained with Stevenel Blue and picrofuchsine and a serial section stained for tartrate-specific acid phosphatase-positive (TRAP) osteoclasts (Acid Phosphatase kit, Sigma-Aldrich, Steinheim, Germany). Using a magnification of 20×, the histomorphometric analysis of each calvaria cap was performed on the most central section and on four adjacent sections. The region of interest was defined as previously recommended [4]. The sagittal suture area (SSA) was determined by tracing the area of soft tissue between the parietal bones. It included resorption pits on the superior surface of the calvaria visible in the same field as the midline suture. To determine bone thickness, sections were divided using a digital caliper in four 100 μm steps to the left and right sides of the midline suture respectively. The measurements were expressed as a percentage of the mean ratio of bone thickness to the total tissue thickness (BT/TT) of the nine regional measurements in the five adjacent sections. To quantify bone loss, results were also expressed as a percentage of the mean difference of BT/TT (Δ(BT/TT)) between sham-operated and particle-implanted animals, in normal controls, sham-OVX, OVX, and OVX + E mice groups. Osteoclasts were identified as large multinucleated cells located on the bone perimeter within a resorption lacuna. Their localization was confirmed in a serial section stained for tartrate-specific acid phosphatase. The osteoclast number was measured in the region of interest of the five consecutive calvaria sections.

### Calvariae culture

Calvariae were removed *en bloc *under sterile conditions from five animals per group randomly assigned for culture. Each calvaria was placed into one well of 12 wells/plate and cultured with 1 ml serum-and phenol-free Dulbecco's modified Eagle's medium (DMEM) with glutamine (Invitrogen, Paisley, UK), and 1% penicillin and streptomycin at 37°C and 5% CO2 for 24 h. Twenty four hours later, the culture medium was collected and stored at -80°C for the assay of IL-1β, IL-6, TNF-α and RANKL secretion. Then, calvariae were calcinated and ashes weighted to normalize the production of cytokines.

### ELISA for IL-1β, IL-6, TNF-α and RANKL detection

To measure IL-6 concentration in serum, blood was obtained by cardiac puncture before death, and collected in heparinized tubes. Blood samples were then centrifuged at 3,000 × g for 10 minutes, aliquoted and frozen at -80°C until assayed. Serum measurements of IL-6 were performed using quantitative, noncompetitive, sandwich enzyme-linked immunosorbent assay kit for detecting mouse IL-6 (Quantikine, R&D Systems Europe, Ltd, Abingdon, UK). Similarly, ELISA kits for detecting mouse IL-6, IL-1β, TNF-α and RANKL (R&D Systems) were employed to measure cytokines released into organ culture supernatant. The detection limits of the assay were 1.6 pg/mL for IL-6, 3 pg/mL for IL-1β, 5.1 pg/mL for TNF-α, and 5 pg/mL for RANKL. Cytokines levels lower than the detection limit were considered to be 0 pg/ml. Absorbance was read in an ELISA reader (Micro-Quant, Bio-Tek Instruments, Colmar, France) at 490 and 540 nm as per manufacturer's instructions.

### Quantitative real-time polymerase chain reaction

Calvariae were removed from culture medium, and immediately snap-frozen in liquid nitrogen, and pulverized to powder in a stainless steel mortar. RNA was extracted with 1 mL Trizol^® ^reagent (Invitrogen, Carlsbad, CA, USA), using the manufacturer's protocol. Quantity and purity of RNA was determined by absorbance on a NanoDrop™ 1000 Spectrophotometer (Labtech France, Palaiseau, France) at 260 and 280 nm. All samples had ratios >1.7 and were accepted for analysis.

Samples of total RNA were reverse-transcribed using the first-strand synthesis kit of SuperScript™ II Reverse Transcriptase (Invitrogen). Quantitative gene expression analyses were carried out using Real-time PCR by means of the TaqMan™ Gene Expression Assays Protocol (mouse RANK: Mm00437135_m1, mouse RANKL: Mm01313943_m1, mouse OPG: Mm01205928_m1, and human 18S rRNA: Hs99999901_s1; Applied Biosystems, Foster City, CA, USA) and the iCycler thermal cycler RT-PCR Detection System, coupled to MyiQ™ Single-Color software (Bio-Rad, Hercules, CA, USA). The absolute number of gene copies was normalized using 18s rRNA and the relative quantification of the genes was calculated using the "comparative *C*_*T *_(threshold cycle) method" as per manufacturer's instructions (Applied Biosystems).

### Data analysis

All values are presented as means ± SEM. Quantitative parameters obtained from micro-CT (BV and Tb.Th) were analyzed by two-tailed Student's *t*-test within each group. Comparisons across unpaired groups were made according to the Mann-Whitney U test. Data concerning osteoclast number, sagittal suture area, and bone thickness were initially analyzed using a one-way analysis of variance (ANOVA). *Post hoc *comparison between groups used the Fisher's protected least significant difference. Two-way ANOVA was performed to determine whether there was a significant effect of either polyethylene particles or ovariectomy or oestrogen supplementation on osteoclast number, sagittal suture area and bone thickness. Cytokines concentrations and mRNAs expression were analyzed using one-way ANOVA followed by the Bonferroni/Dunnet's test. The level of statistical significance was set at *P *< 0.05.

## Results

### Changes after OVX

All mice had increased body-weight by the end of the experiment. The final body-weight gain was +18% in the OVX group as compared to +6.8% (*P *< 0.0001), +3.5% (*P *< 0.0001), and +11% (*P *= 0.0001) in normal controls, sham-OVX mice, and OVX+E mice, respectively.

At sacrifice, atrophy of uterine tissue was noted, indicating the effectiveness of oestrogen privation following ovariectomy. There was a significant difference in uterine weight in OVX mice group (mean, 24.5 ± 3.6 mg), as compared to normal control animals (mean, 107.9 ± 12 mg, *P *< 0.0001), sham-OVX mice (mean, 86.4 ± 17 mg, *P *< 0.0001) and OVX+E mice (mean, 80.2 ± 10.9 mg, *P *< 0.0001). In contrast, uterine weight was notably less altered after oestrogen supplementation in OVX animals as compared to normal controls (80.2 ± 10.9 mg versus 107.9 ± 12 mg, *P *= 0.002).

### Micro-CT evaluation of osteolysis

**Figure 1:**
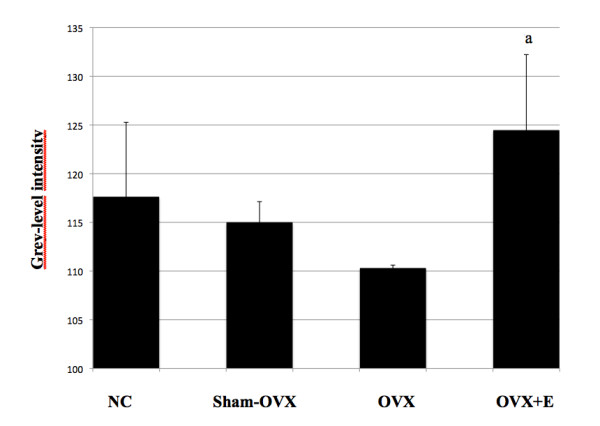
**Grey-level intensity in entire calvariae, as assessed by 3D micro-CT**. In the absence of PE particles, mean grey-level values decreased in ovariectomized (OVX) mice as compared to normal controls (NC) and Sham-OVX mice. Of note is the significant increase of grey-level intensity in calvarial bone tissue of OVX animals supplemented with oestrogen (E), as compared to OVX mice. Grey-level values were obtained using CTAn software. The data are presented as means ± standard error of the mean (**a **indicates *P *< 0.05, as compared to OVX mice, using the Mann-Whitney U test).

**Figure 2:**
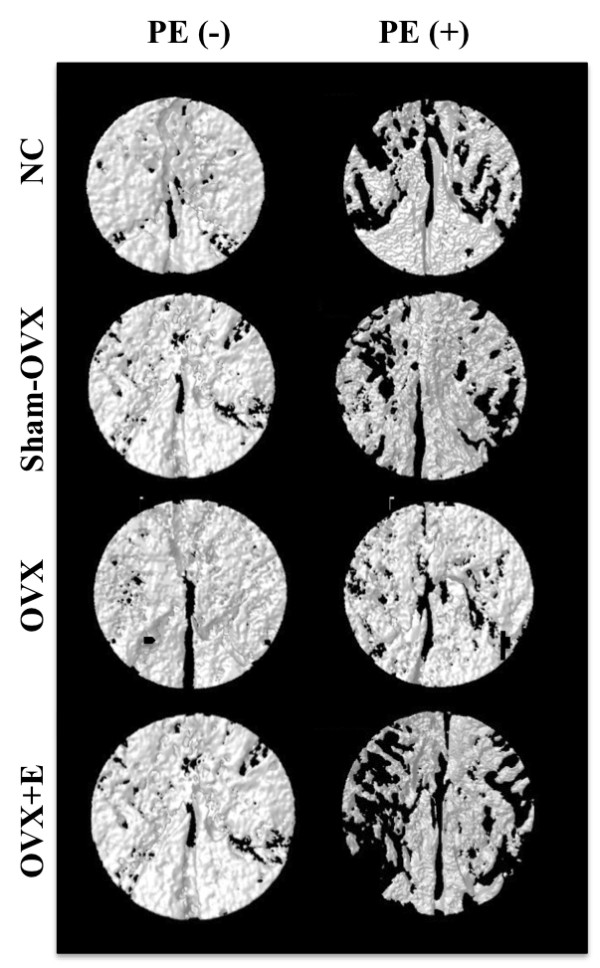
**Effect of polyethylene particles on calvariae, as assessed by longitudinal 3D micro-CT**. Representative reconstructed images of the calvarial volume of interest (VOI), two weeks after sham-implantation (PE(-)) or PE particles implantation (PE(+)) in different mice groups are shown (*n *= 7 per group). Of note is the consistent extent of bone resorption area following particles implantation in normal controls (NC), sham ovariectomized (OVX) mice and OVX animals supplemented with oestrogen (E), while bone resorption area appears limited in OVX animals implanted with particles.

**Figure 3:**
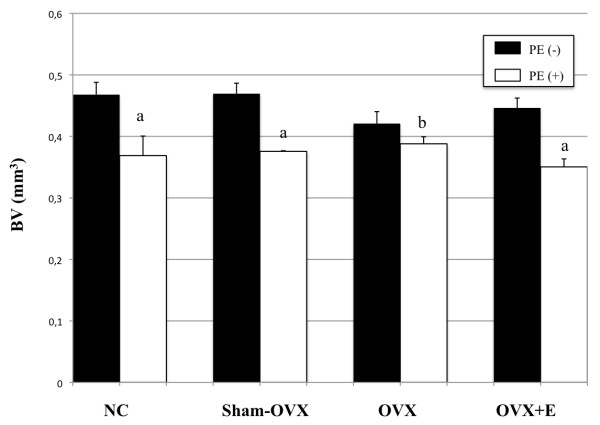
**Effect of polyethylene particles on bone volume (BV), as assessed by longitudinal 3D micro-CT**. Bone volume consistently decreased in all mice groups. However, bone loss was significantly lower in ovariectomized (OVX) mice as compared to normal controls (NC), sham-OVX mice and OVX animals supplemented with oestrogen (E). Bone volume was quantified using CTAn software. The data are presented as means ± standard error of the mean (**a **indicates *P *< 0.005; **b **indicates *P *< 0.05, compared to internal control, using Student's *t*-test).

**Figure 4:**
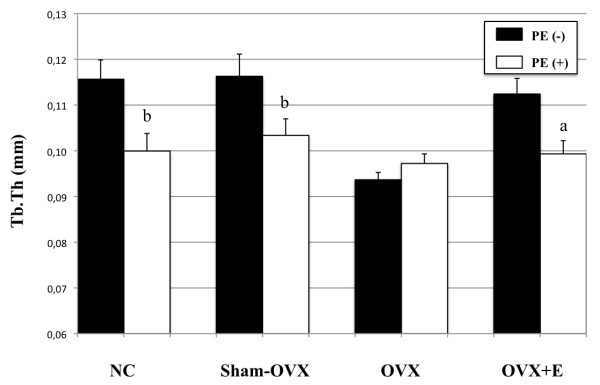
**Effect of polyethylene particles on trabecular thickness (Tb.Th), as assessed by longitudinal 3D micro-CT**. Trabecular thickness was not altered by the presence of particles in ovariectomized (OVX) mice, as opposed to the other mice groups. Trabecular thickness was quantified using CTAn software. The data are presented as means ± standard error of the mean (**a **indicates *P *< 0.005; **b **indicates *P *< 0.05, compared to internal control, using Student's *t*-test).

Ovariectomy induced a consistent, although non-significant, decrease in mean grey-level intensity in calvariae without PE particles as compared to normal controls (Figure 1). Specifically, mean grey-level values were 117.6 ± 7.7 (range, 107 to 48), 115 ± 2 (range, 107 to 120) and 110 ± 0.3 (range, 109 to 111), in normal controls, sham-OVX mice and OVX mice, respectively. In contrast, grey-level intensity significantly increased in OVX animals after oestrogen supplementation (124 ± 7.8), as compared to OVX mice (*P *= 0.02), while it was similar to normal controls (*{ P *= 0.22) and sham-OVX mice (*P *= 0.07). The presence of PE particles induced profound changes in calvarial bone microarchitecture in all groups (Figure 2). In particle-implanted animals, BV significantly decreased within the VOI as compared to sham-implanted animals in normal control group (*P *= 0.004), in sham-OVX group (*P *= 0.003), in OVX group (*P *= 0.04) and in OVX+E group (*P *= 0.001) (Figure 3). However, decrease in BV following particle implantation was consistently less marked in the OVX group (-7.8%, as compared to -21.2% in normal control group, *P *= 0.04, to -19.9% in sham-OVX group, *P *= 0.003, and to -21.4% in OVX+E group, *P *= 0.005). Polyethylene particles did not produce any significant decrease in bone thickness (Tb.Th) in OVX mice (+3.8% as compared to sham-implanted OVX mice, *P *= 0.08), in contrast with normal controls (-13.6% as compared to sham-implanted normal controls, *P *= 0.01), sham-OVX mice (-11.1% as compared to sham-implanted sham-OVX mice, *P *= 0.008), and OVX+E group (-11.6% as compared to sham-implanted OVX+E mice, *P *= 0.005) (Figure 4).

### Histology

**Figure 5:**
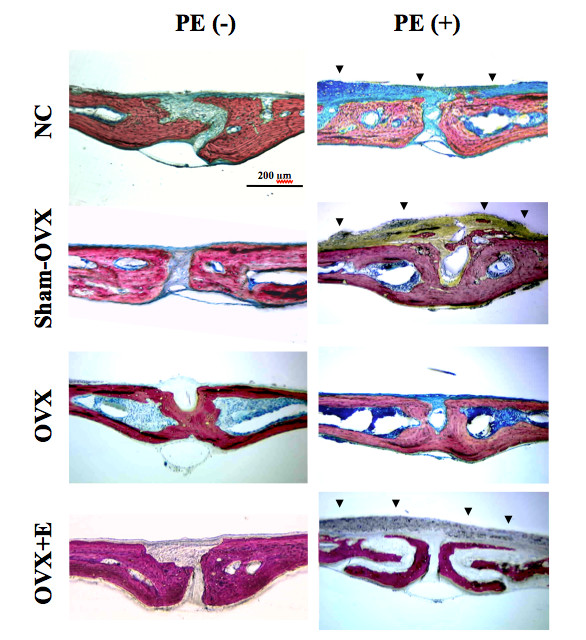
**Effect of polyethylene particles on calvariae, as assessed by histology**. Representative stained calvarial histology (Stevenel Blue, picrofuschine) is presented at ×20 original magnification (*n *= 7 per group). Polyethylene particles induced osteolysis, characterized by a consistent decrease in calvarial bone thickness and an inflammatory response (arrowheads) in normal controls (NC), sham-ovariectomized (OVX) mice and OVX mice supplemented with oestrogen (E), while it was moderate in OVX mice. PE(-): sham-implanted; PE(+): PE-implanted.

Histological sections showed a consistent erosion of the calvarial bone, associated to fibrous and granulomatous scar tissue layer on the periosteal side of the calvaria in particle-implanted mice, in all groups. The most dramatic lesions were observed when PE particles were implanted in the OVX+E group (Figure 5). However, the tissue response to particles appeared limited in OVX animals without E supplementation. Using polarized light, many particles were found intracellularly within macrophages and foreign-body giant cells. TRAP staining confirmed the presence of osteoclasts located in resorptive lacunae.

Histomorphometric data are shown in Table 2. One-way ANOVA revealed a significant difference for the sagittal suture area (*P *< 0.0001). The presence of PE particles induced a significant increase in SSA in normal control group (*P *< 0.001), in sham-OVX group (*P *< 0.001), in OVX group (*P *< 0.001), and in OVX+E group (*P *< 0.0001). No significant difference was found in SSA between sham-implanted normal controls group and sham-implanted OVX group (*P *= 0.9), between PE-implanted normal control mice and PE-implanted OVX mice (*P *= 0.99), and between sham-implanted normal control mice and sham-implanted OVX+E mice (*P *= 1). Two-way ANOVA revealed a significant effect of PE particles on SSA (*P *< 0.0001). There was no independent effect of OVX on SSA (*P *= 1) in the absence of particles. One-way ANOVA revealed a significant difference for osteoclast number (*P *< 0.0001). The presence of PE particles induced a consistent increase in osteoclast number in normal control group (*P *< 0.0001), in sham-OVX group (*P *< 0.001), and in OVX+E group (*P *< 0.0001), but not in OVX group (*P *= 0.99). There was also a significant effect of ovariectomy on osteoclast number (*P *= 0.047) in the absence of particles.

**Table 2:** Histomorphometric results for each mice group

	**Normal control group**		**Sham-OVX group**		**OVX group**			
					
					**OVX + Vehicle**		**OVX+E**	
	PE (-)	PE (+)	PE (-)	PE (+)	PE (-)	PE (+)	PE (-)	PE (+)
**SSA (mm^2^)**	0.036 ± 0.008 (0.008 to .076)	0.10 ± 0.04 (0.05 to 0.33)	0.034 ± 0.007 (0.008 to 0.07)	0.093 ± 0.03 (0.05 to 0.34)	0.026 ± 0.01 (0.006 to 0.1)	0.09 ± 0.04 (0.01 to 0.35)	0.038 ± 0.01 (0.01 to 0.11)	0.20 ± 0.05 (0.07 to 0.46)
**BT/TT**	60 ± 4.7% (42% to 80%)	48 ± 5.3% (23% to 73%)	62 ± 4.6% (49% to 80%)	45 ± 4% (23% to 56%)	55 ± 5.2% (35% to 73%)	49 ± 4.9% (26% to 65%)	54 ± 5.5% (31% to 73%)	37 ± 5.1% (17% to 55%)
**Δ(BT/TT)**	-12 ± 5.3% (-37% to 12%)		-16 ± 4.5% (-39% to 5%)		-6.7 ± 4.9% (-30% to 9%)		-17 ± 5% (-37% to 1.2%)	
**Oc Nb**	0.31 ± 0.3 (0 to 2)	2.2 ± 0.7 (0 to 5)	0.42 ± 0.3 (0 to 2)	2.5 ± 0.7 (0 to 5)	0.65 ± 0.4 (0 to 3)	0.7 ± 0.5 (0 to 4)	0.27 ± 0.2 (0 to 1)	2.1 ± 0.8 (0 to 6)

Values are expressed as mean ± standard error of the mean (extremes). BT, bone thickness; E, oestrogen; Oc Nb, osteoclast number; OVX, ovariectomy; PE, polyethylene; PE(-), sham-implanted; PE(+), PE-implanted; SSA, sagittal suture area; TT, tissue thickness.

One-way ANOVA showed a significant difference for bone thickness (*P *< 0.0001). Implantation of PE particles resulted in a marked decrease in BT/TT in normal control group (*P *= 0.001), in sham-OVX group (*P *< 0.001), and in OVX+E group (*P *< 0.0001). However, the presence of PE particles did not influence BT/TT in OVX group (*P *= 0.088). Also, two-way ANOVA showed an independent effect of ovariectomy on BT/TT (*P *< 0.001) in the absence of particles.

### Serum levels of IL-6

**Figure 6:**
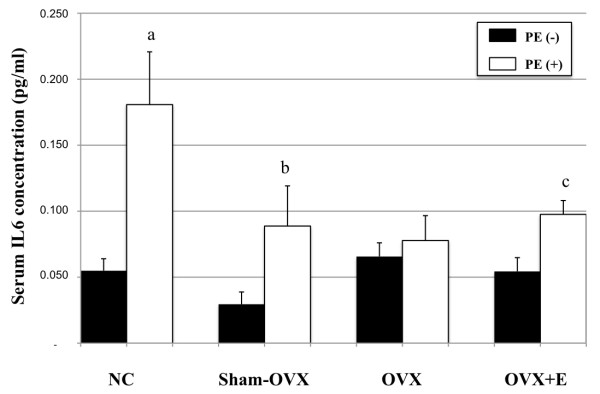
**Effect of polyethylene particles on serum levels of IL-6 in each mice group**. Blood was obtained by cardiac puncture 14 days after surgery (*n *= 5 per group). Serum levels of IL-6 were determined by ELISA. Results are presented as mean ± standard error of the mean (**a **indicates *P *< 0.01; **b **indicates *P *= 0.01; **c **indicates *P *< 0.005, compared to internal control).

Serum levels of IL-6 significantly increased after PE particles implantation in all mice groups, as compared with sham-implanted internal controls (*P *= 0.008 in normal control group, *P *= 0.01 in sham-OVX mice, and *P *= 0.02 in OVX+E mice), except in OVX mice group (*P *= 0.57) (Figure 6). Ovariectomy did not significantly increase serum IL-6 concentrations in the absence of particle implantation (sham-implanted OVX group versus sham-implanted normal controls, *P *= 0.78, and sham-implanted OVX+E group versus sham-implanted normal control mice, *P *= 0.84).

### Production of cytokines in organ culture

**Figure 7:**
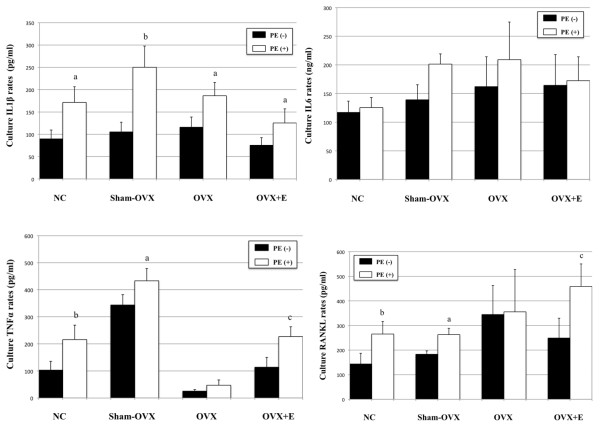
**Effect of polyethylene particles on the local expression of IL-1 β, IL-6, TNF-α and RANKL (ELISA)**. Cytokines concentrations were determined after 24 hours in culture medium by ELISA (*n *= 5 per group). Results are presented as mean ± standard error of the mean. **a **indicates *P *< 0.05, **b **indicates *P *< 0.005, **c **indicates *P *= 0.001, compared to internal control. IL-1 β, interleukin 1 β; IL-6, interleukin-6; TNF-α, tumor necrosis factor-α; RANKL, receptor activator of the nuclear factor κB ligand.

The presence of PE particles induced a significant increase in IL-1 β concentration in all mice groups (Figure 7). However, particles implantation did not significantly influence IL-6 media rates as compared to internal controls (Figure 7). In OVX mice without particles, the levels of TNF-α were significantly lower than in normal controls (*P *= 0.02). In addition, in this group, PE particles did not increase the rates of TNF-α (*P *= 1), which remained considerably lower than in normal controls and in OVX+E mice with PE particles (*P *= 0.002 and *P *= 0.002, respectively) (Figure 7). Significant differences in RANKL basal release (absence of particles implantation) were found between OVX mice and each other mice groups (normal controls, *P *= 0.047, sham-OVX mice, *P *= 0.016, OVX+E mice, *P *= 0.02). Noteworthy, RANKL local production appeared to be stimulated by PE particles in normal controls, in sham-OVX mice, and in OVX+E mice, while in OVX group, no significant modification in RANKL rates could be detected following particles implantation (*P *= 0.95) (Figure 7).

### Quantitation of RANKL and OPG mRNA expressions in calvariae

**Figure 8:**
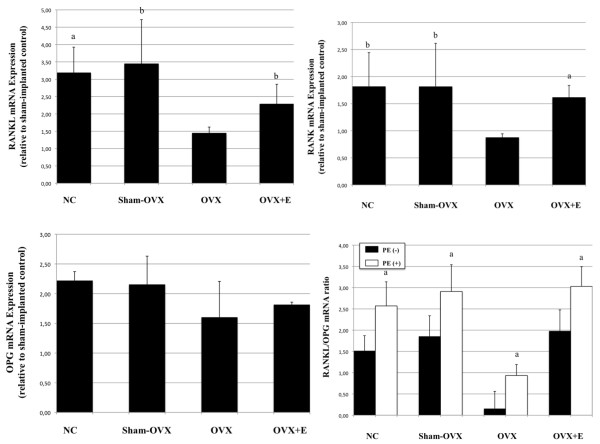
**Effect of polyethylene particles on the expression of RANKL, RANK and OPG in bone calvariae**. Quantitative real-time PCR analysis were performed 24 hours after calvariae culture (*n *= 5 per group). For every sample, experiments were carried out in triplicate and results are presented as mean ± standard error of the mean. **a **indicates *P *< 0.05, compared to sham-implanted internal controls. OPG, osteoprotegerin; RANK, receptor activator of the nuclear factor κB; RANKL, RANK ligand.

As shown in Figure 8, the combined effect of ovariectomy and PE particles implantation stimulated RANKL mRNA expression in calvariae, in all mice groups. However, PE-implanted calvariae in normal controls, sham-OVX and OVX+E mice expressed RANKL mRNA at levels more than twice higher than OVX mice. OPG mRNA expression was not significantly different between groups (Figure 8). As a result, the RANKL/OPG mRNA ratio significantly increased in all mice groups after PE implantation, but appeared consistently downregulated in OVX mice (Figure 8). The RANKL/OPG mRNA ratio significantly differed between OVX mice and each other mice group in the absence of particles (*P *= 0.047 as compared to normal controls, *P *= 0.034 as compared to sham-OVX mice, and *P *= 0.049 as compared to OVX+E mice). In addition, no significant difference could be observed in the basal RANKL/OPG mRNA ratio between normal controls and OVX+E mice, indicating that E supplementation reestablished RANKL mRNA expression relative to OPG mRNA.

## Discussion

In a recent work [6], we have shown that experimental stimulation of bone turnover by OVX resulted in a paradoxical decrease in particle-induced osteolysis in the murine calvaria model. To test the hypothesis that oestrogen-deficiency induced by OVX modulated the bone response to particulate debris, we supplemented PE particle-implanted OVX mice with 17β-estradiol. Then, we evaluated osteolysis and the involvement of the pro-inflammatory cytokines IL-6, IL-1β, TNF-α, and RANKL.

Our experiments show that the presence of PE particles induced an osteolytic reaction in bone calvaria in normal controls, in sham-OVX mice and in OVX+E mice, as illustrated by an extensive bone resorption and an intense inflammatory reaction involving both bone and periosteal tissue. In contrast, this process was considerably attenuated in OVX mice, with almost no modification in trabecular thickness, osteoclast number and periosteum thickness following PE implantation. In addition, Bone volume appeared consistently less altered following particles implantation, as compared to normal controls and OVX mice supplemented with oestrogen. Taken together, these data suggest that oestrogen deficiency influenced positively bone response to particulate debris. This finding appears paradoxical as clinical trials [14-16] and experimental studies [17,18] have demonstrated the protective effects of oestrogen against inflammatory processes, such as rheumatoid arthritis (RA). However, the relationship between oestrogen and inflammation is controversial and might be related to a different response of bone and inflammation cells. Although it is admitted that oestrogen provides a protection against inflammation, it is also known that female patients are more prone to develop inflammatory diseases such as RA [19]. This point illustrates the dual action of oestrogen, both anti- and pro-inflammatory.

Numerous cellular and molecular mechanisms leading to bone resorption are common to osteolysis and oestrogen deficiency pathways. Cytokines such as IL-1, IL6, TNF-α, and RANKL are up-regulated in both bone-resorptive situations [20-24]. Differentiation of RANKL-primed osteoclasts precursors, activation of mature osteoclasts, and osteoclasts survival are enhanced by TNF-α [25]. Zhang *et al. *[26] showed that TNF-α and RANKL act synergistically on osteoclastogenesis *in vitro*. The role of TNF-α in OVX-induced bone loss is well documented [20,27,28]. Collectively, these studies suggest that up-regulation of TNF-producing cells in the bone marrow is a mechanism by which oestrogen deficiency induces bone loss. Indeed, functional blockade of TNF-α in mice leads to protection from OVX-induced bone loss [27]. Accordingly, we found that TNF-α local production was consistently altered in oestrogen deficient mice, suggesting its involvement in limited osteoclastogenesis in this group.

In our experiments, local release of pro-inflammatory cytokines IL1- β and IL-6 appeared heterogeneous. On one hand, we found that pro-inflammatory cytokine IL-1 β was stimulated by PE particles in all groups. On the other hand, IL-6 local rates were not increased in the presence of PE particles in any group. Indeed, it has been suggested that the involvement of pro cytokines *in vivo *may be difficult to demonstrate due to compensatory process [29], and to the delayed time course of release after particle implantation. Conversely, serum IL-6 was stimulated by PE implantation in normal controls, sham-OVX and E-supplemented OVX mice, whereas no significant change occurred in OVX mice. This point correlated well with the degree of osteolytic reaction combining bone resorption and periosteal inflammatory granuloma. The role of IL-6 as a stimulator of bone resorption in post-menopausal osteoporosis is well-known [30]. IL-6 promotes bone resorption by affecting osteoclast differentiation and activity [31]. Our results are in line with clinical data showing that serum levels of IL-6 were consistently increased in patients with loosened hip prosthesis [32]. However, authors failed to correlate serum levels of IL-6 and the volume of osteolysis [33]. These discrepancies suggest that serum cytokine levels may be influenced by various factors, such as cytokine release from other organs, and cytokine clearance rates from the joint. Taken together with our findings, we speculate that serum and local IL-6 changes may be involved in the mechanism underlying the reduction of particle-induced osteolysis observed in OVX mice. Thus, the hormonal status appears as a key factor influencing serum IL-6 levels to be considered in further clinical studies.

We observed that the combination of two bone-resorptive stimuli resulted in a down-regulation of local pro-resorptive cytokines TNF-α and RANKL, in parallel with a limitation of bone loss. The latter appeared as a consequence of decreased osteoclast activity and/or recruitment and/or accelerated apoptosis. We assume this process is regulated by the RANK/RANKL/OPG pathway, synergistically with TNF-α secretion, since RANKL is required for osteoclasts differentiation, survival and activity [21]. Pro-resorptive cytokine production could have been altered at the cellular level. Hence, particle-induced osteolysis and postmenopausal osteoporosis represent pro-inflammatory states with increased inflammatory cytokines and activated T cells [34]. Cytokines produced or regulated by T cells can enhance osteoclast formation by increasing the RANKL/OPG ratio [35]. Oestrogen deficiency also increases the RANKL/OPG ratio through increases in pro-inflammatory effectors, including IL-1β, IL-6, IL-11, TNF-α, M-CSF and PGE [36]. RANKL expression was observed in osteoblasts/stromal cells and bone marrow in postmenopausal women. The molecular basis of the mechanism by which oestrogen deficiency induces accelerated osteoclastogenesis is not yet elucidated [37]. In mice, the selective ablation of oestrogen receptor in mature osteoclasts mimicked an osteoporotic bone phenotype [24], suggesting that oestrogen directly regulates the life span of differentiated osteoclasts, independently of an inflammatory pathway.

We hypothesize that the paradoxical decrease in bone response to PE particles in OVX mice could be driven by an innate protective mechanism that ultimately attenuated focal bone resorption. Hence, a tight control of innate immunity is essential because a disproportionate immune response may considerably compound morbidity. Recent studies suggested the involvement of T cells not only in pathogen clearance but also in regulating adaptative and innate immunity [38]. Recently, Guarda *et al*. [39] reported that excessive inflammation was suppressed by effector and memory T cells, via the blockade of mature IL-1β secretion, suggesting a possible mechanism for the control of excessive inflammatory responses.

We recognize limitations may have affected our findings. First, calvarial bone tissue considerably differs from long bone tissue with respect to cells, matrix composition and mineralization. Osteoclasts activity has been shown to be highly variable, depending on bone-site origin [40,41]. Specifically, it was reported that proteolytic enzymes used for bone matrix digestion [40,42], expression of TRAP enzyme, and size of osteoclasts originated from calvarial bone differed from those present in long bones [41,43]. Additionally, van den Bos *et al. *[44] reported that calvarial bone exhibited differences in matrix composition compared to long bone, including degree of mineralization, amounts of collagen, osteoglycin, and pigment epithelium derived factor. These data together indicate a putative difference in bone resorption characteristics in calvaria. Although the murine calvarial model has been extensively used to evaluate particle-induced osteolysis [4-6], the clinical relevance of our data should be considered with caution. Second, the osteolysis murine model does not fully reproduce the loading conditions of a prosthetic implant subjected to loosening. The consequences of OVX on mouse calvarial bone have been poorly studied. However, our observations revealed that basal calvarial bone thickness was significantly reduced in OVX mice, confirming previous findings [10,45]. This is consistent with bone resorption of the skull and subsequent parietal bone thinning observed in postmenopausal osteoporotic women [46]. Therefore, the current experimental protocol likely mimicked comparable cellular and biologic bone responses in OVX mice that occur in the early postmenopausal period. In the present work, we did not evaluate cytokines local rates in a time-dependant fashion after particles implantation. Hence, pro-inflammatory cytokines are known to be highly labile and, subsequently, their local and serum concentrations may vary with time. However, we limited our experiments to the time period preceding the onset of theoretical substantial bone repair. Therefore, it is likely that our cytokines and mRNA evaluations are compatible with bone cellular response tested in our model.

## Conclusions

The present study analysed the consequences of oestrogen deprivation in a murine model of particle-induced osteolysis. We demonstrated that oestrogen deficiency significantly attenuated osteolytic response to PE particles. This phenomenon was associated with a down-regulation of pro-inflammatory and pro-resorptive cytokines TNF-α and RANKL, together with an absence of increase of serum IL-6. We favour the alternative that the combination of two bone-resorptive phenomenon altered cytokines production, possibly via the implication of the periosteum in this mice model.

## Abbreviations

BT: bone thickness; BV: bone volume; CT: computed tomography; E: oestrogen; ELISA: enzyme-linked immunosorbent assay; IL-1β: interleukin-1β; IL-6: interleukin-6; LAL: Limulus Amebocyte Lysate; OPG: osteoprotegerin; OVX: ovariectomy; PE: polyethylene; RANKL: RANK ligand; RANKL: receptor activator of the nuclear factor κB; RT-PCR: real-time polymerase chain reaction; SSA: sagittal suture area; Tb.Th: trabecular thickness; TNF-α: tumor necrosis factor-α; TRAP: tartrate-specific acid phosphatase-positive; TT: tissue thickness; VOI: volume of interest.

## Competing interests

The authors declare that they have no competing interests.

## Authors' contributions

CN carried out the histological experiments and analyses and drafted the manuscript. JL carried out the cytokines measurements and RT-PCR. AM carried out micro-CT analyses. All other authors were involved in the design of the study, interpretation of the data and revision of the manuscript. All authors read and approved the final manuscript.
